# Effects of Curcumin and Tetracycline Gel on Experimental Induced Periodontitis as an Anti-Inflammatory, Osteogenesis Promoter and Enhanced Bone Density through Altered Iron Levels: Histopathological Study

**DOI:** 10.3390/antibiotics11040521

**Published:** 2022-04-13

**Authors:** Chenar Anwar Mohammad, Khadeeja Mohammed Ali, Rafal AbdulRazaq Al-Rawi, Sarhang Sarwat Gul

**Affiliations:** 1 Department of Periodontology, College of Dentistry, Hawler Medical University, Erbil 44001, Iraq; chenaranwar0@gmail.com (C.A.M.); drkhadij@yahoo.com (K.M.A.); 2 Department of Clinical Analysis, College of Pharmacy, Hawler Medical University, Erbil 44001, Iraq; rafal.alrawi@hmu.edu.krd; 3 Department of Periodontics, College of Dentistry, University of Sulaimani, Sulaymaniyah 46001, Iraq

**Keywords:** periodontitis, rats, tetracycline, curcumin, scaling and root planing, inflammatory markers, osteogenesis, iron level

## Abstract

Adjunctive use of antimicrobials with scaling and root planing (SRP) is necessary to better eradicate dental biofilm. Tetracycline (T) is the most commonly used antimicrobial; however, it has limitations. This study evaluates the effect of curcumin (CU) as adjunct to SRP on inflammatory markers, collagen fiber deposition, and altered iron level. A total of 32 Wistar rats were divided into five groups: no experimental periodontitis (healthy control), experimental periodontitis (EPD), EPD treated with SRP alone (SRP), EPD treated with SRP+T (SRP+T), and EPD treated with SRP+CU (SRP+CU). After 2 and 4 weeks of treatment, tissue samples were assessed by hematoxylin and eosin, and special stains (Perls’ stain and Masson’s Trichrome) for counting of inflammatory cells, angiogenesis, collagen fibers, and iron deposition. Significant reductions in inflammatory cells infiltration and alveolar bone resorption with angiogenesis and collagen fibers deposition were detected after 2 and 4 weeks in both SRP+T and SRP+CU groups. SRP+CU resulted in a significant reduction in osteoclast numbers (week 2) and iron deposition (week 4) in bone trabeculae as compared to SRP and SRP+T groups. The adjunctive use of CU showed comparable results to T in the reduction in inflammation and bone resorption. Furthermore, CU has potential osteogenesis and healing effects.

## 1. Introduction

Periodontitis is one of the most prevalent diseases affecting humankind, resulting in the destruction of the periodontal tissues by the interaction of periodontopathogenic microorganisms and the host’s immune response [1]. According to the new classification of periodontal diseases, periodontitis can be defined based on the presence of interdental clinical attachment loss, probing pocket depth, and alveolar bone loss [2]. Many studies have used experimental animals to investigate the pathogenesis of periodontal disease, and placing ligatures on the teeth has been suggested to make experimental periodontitis occur more quickly than happens naturally [3]. A clinical and histopathological analysis showed that a “ligature” model of periodontitis in rats has several advantages, such as rapid induction of disease in 14 days, pronounced clinical inflammation of periodontal tissues, and advanced resorption of the alveolar bone [4].

Periodontitis has been linked to serious systemic diseases such as atherosclerosis, cardiovascular diseases, stroke, rheumatoid arthritis, Alzheimer’s disease, chronic kidney disease or malignant neoplasia. Periodontal therapy depends on the reduction of pathogenic microbiota by scaling and root planing (SRP), and it has shown to have impact on glycemic control in individuals with type 2 diabetes mellitus and periodontitis and the prevention of respiratory infections [3]. However, mechanical therapy alone may be unsuccessful in eliminating pathogenic bacteria that are within the soft tissue and in areas inaccessible to periodontal instrumentation, such as the furcation area and root concavities. Because of these limitations, several studies have attempted to demonstrate clinical improvement with the administration of adjunctive systemic or local antimicrobial medications [5,6,7]. The local administration of adjunctive antimicrobial agents to the periodontal pockets showed better clinical improvements, especially in the sites that did not respond to the mechanical periodontal therapy alone [8].

Substantivity is the most important property for an antimicrobial when used by means of local administration [9]. The local delivery of antimicrobials to the periodontal pockets has the benefit of maintaining adequate concentration of the drugs at the desired site while minimizing the exposure of the body to the drug. The antimicrobials commonly used as local drug delivery agents are tetracycline (T), ofloxacin, and clindamycin [10]. 

Tetracycline and its derivatives, such as doxycycline and minocycline, are the most commonly used adjunctive antimicrobials in the treatment of periodontal diseases. Tetracycline also binds to the root surfaces and can be released in active form over extended periods of time. The sub-lethal concentration of T reduces adherence and co-aggregation properties of a number of periodontal disease-associated bacteria including *Porphyromonas gingivalis* and *Prevotella intermedia* [11], inhibits collagenase [12], as well as increases fibroblast attachment to dental structures when associated with fibronectin [13]. However, these antimicrobials have undesirable side effects and limitations [7,10]. Therefore, herbal plants are used as an alternative to these antimicrobials as they are relatively safe. Among these herbal products, curcumin (CU) is widely investigated as adjunct in the treatment of periodontal diseases. Curcumin has shown potent anti-inflammatory, antibacterial, antioxidant, and astringent properties with significant improvement in gingival health when used for subgingival irrigation [14]. Furthermore, CU has been shown to reduce bone resorption by periodontal disease when used as adjunct to SRP [15]. However, the bone formation effect of CU has not been investigated yet. 

Bone mineralization is mediated by osteoblasts via producing and secreting osteoid matrix for mineralization. Osteoblasts maintain a higher iron requirement for proper differentiation, mineralization, and function. The mechanism of disrupted bone homeostasis during iron deficiency still remains unclear [16]. Therefore, the present study is the first to evaluate the effect of CU as adjunct to SRP on inflammatory markers, collagen fiber deposition, and altered iron level in the treatment of experimental induced periodontitis in rats. 

## 2. Results

### 2.1. Histological Evaluation

#### 2.1.1. Inflammatory Reaction

**Figure 1:**
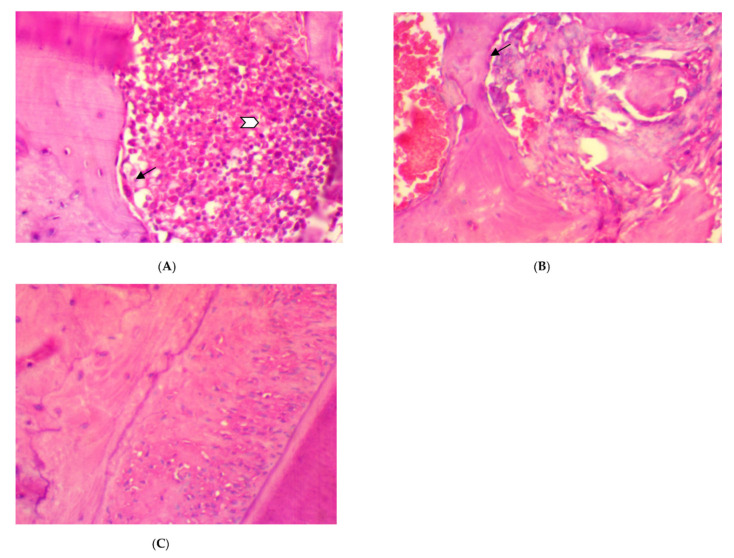
The histopathological pictures of rat periodontal tissue after 14 days ligation in experimental periodontitis group, (**A**,**B**). (**A**) Moderate inflammatory cells in the periodontal ligament (white arrow), active alveolar bone resorption, and osteoclast cells (black arrow) bordering the bone trabeculae. (**B**) Active alveolar bone resorption, osteoclast cells (arrow) bordering the bone trabeculae, and disorientation of periodontal collagen fibers and hemorrhage. (**C**) Histological aspects of rat periodontal tissue in the normal control: normal alveolar bone and well-arranged collagen fibers with no blood vessels congestion or inflammatory cells infiltration (score 0) in the periodontal ligament (H&E ×400).

The histopathological features of periodontal tissue after 14 days ligation in EDP group showed the presence of active alveolar bone resorption, moderate inflammatory cells infiltration (score 2), disorientation of collagen fibers, and congestion of blood vessels (Figure 1A,B) as compared to the control rat group with normal border of alveolar bone, well-arranged collagen fibers with no blood vessels congestion, and inflammatory cells infiltration (score 0) in the periodontal ligament (Figure 1C).

**Figure 2:**
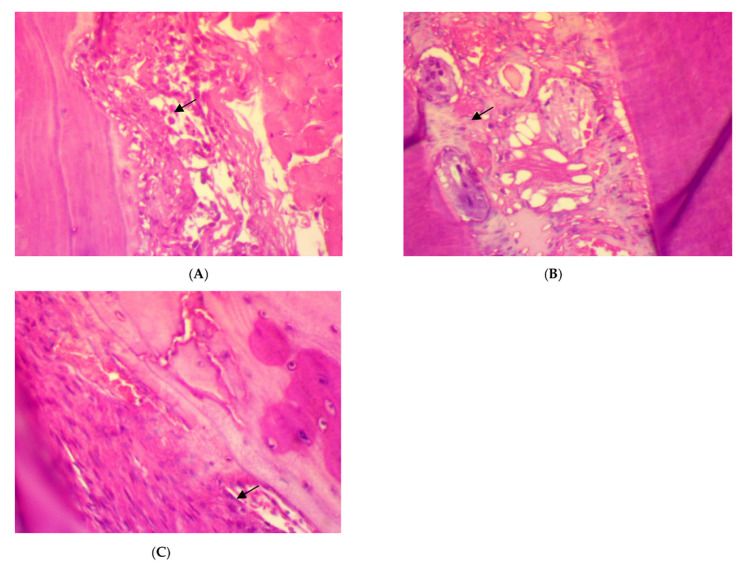
Histopathological features of rat periodontal tissue after two weeks of treatment show: (**A**) mild inflammatory cells infiltrations (arrow) in the periodontal ligament with collagen fibers disorientation, fibroblast cells, and vascular congestion in SRP group. (**B**,**C**) Few inflammatory cells infiltrations (arrow) in the periodontal ligament with less collagen fibers disorientation, fibroblast cells, and vascular congestion in SRP+T (**B**) and SRP+CU groups (**C**) (H&E ×400).

The histopathological features of rat periodontal tissue after two weeks of treatment in the SRP group showed mild inflammatory cells infiltrations (score 1) in the periodontal ligament with immature unorganized collagen fibers, fibroblast cells, and blood vessels congestion. Meanwhile, few inflammatory cells infiltrations (score 0), with less collagen fibers disorientation, fibroblast cells, and vascular congestion with evaluated parameter expression of moderate (score 1) could be seen after two weeks of treatment in both SRP+T and SRP+CU groups (Figure 2). 

**Figure 3:**
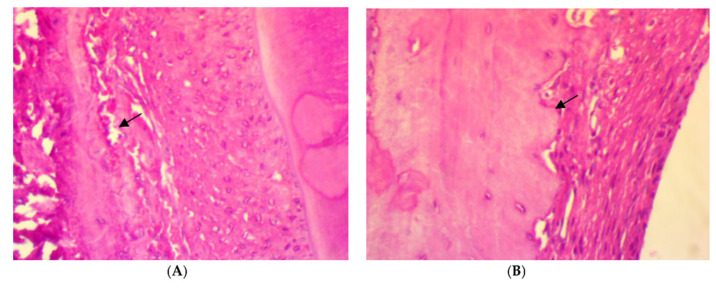

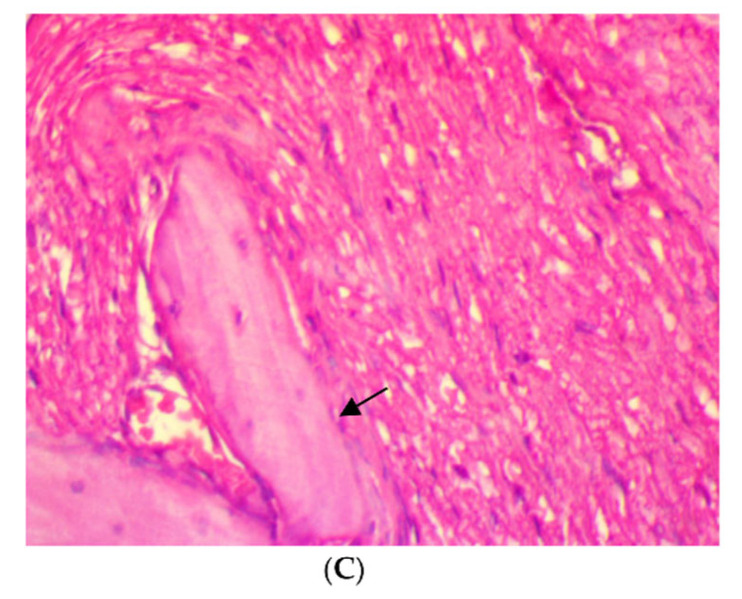
Histopathological features of rat periodontal tissue after a four-week treatment show: (**A**) a few inflammatory cells infiltrations in the periodontal ligament, blood vessels congestion (arrow), fibroblast cells, and osteoblasts bordering the alveolar bone trabeculae (arrow) in SRP group. (**B**,**C**) No inflammatory cells infiltrations in the periodontal ligament, mild blood vessels congestion, well-arranged collagen fibers, spindle-shaped fibroblast cells, and osteoblasts bordering the alveolar bone trabeculae (arrow) in SRP+T (**B**) and SRP+CU (**C**) groups (H&E ×400).

After four weeks of treatment, in SRP group, the histopathological features of the rat periodontal tissue showed the presence of a few inflammatory cells in the periodontal ligament, a moderate amount of collagen fibers, the congestion of blood vessels, fibroblast cells appearing as spindle shapes (parameter expression score of 1), and the appearance of osteoblast cells bordering the alveolar bone trabeculae. Meanwhile, in SRP+T and SRP+CU groups, no inflammatory cells infiltrations (score 0) with only blood vessels congestion, well-oriented mature collagen fibers, fibroblast cells appearing as mature spindle shapes, and strong parameter expression (score 2) could be seen, as well as osteoblast cells bordering the alveolar bone with no inflammatory cells (Figure 3).

#### 2.1.2. Special Stains

**Figure 4:**
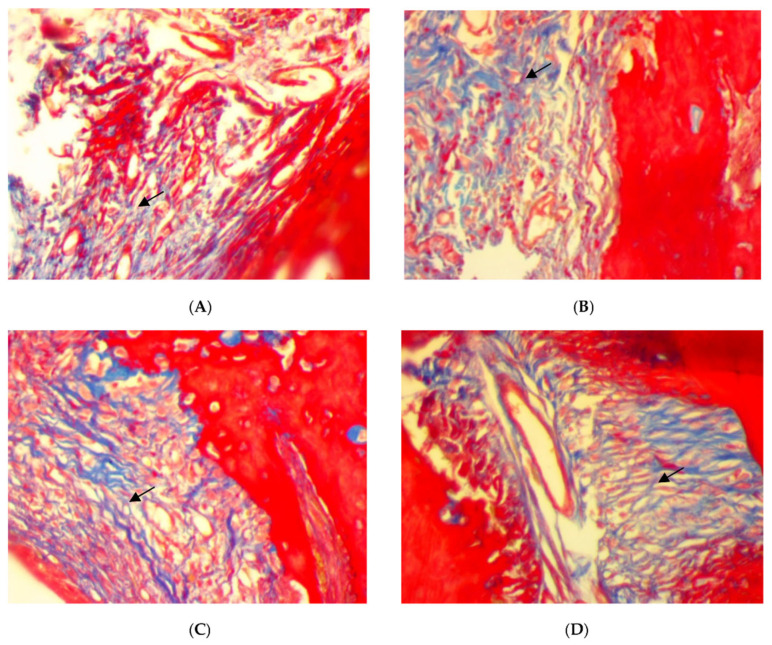
Histopathological features of rat periodontal tissue (**A**) after 2 weeks ligation in EPD group showing disorientation of a few formed immature collagen fibers (arrow). (**B**–**D**) Histopathological features of rat periodontal tissue after 2 weeks treatment show: (**B**) disorientation of a few formed immature collagen fibers (arrow) in SRP group, (**C**,**D**) less collagen fiber disorientation with little remodeling effect, and a moderate amount of collagen fibers, grouped in poorly cohesive beams (arrow) in SRP+T (**C**) and SRP+CU (**D**) groups (Trichrome ×400).

Trichrome stain for rat periodontal tissue after two weeks of ligation in EDP group showed the disorientation of a few formed immature collagen fibers (score 0) (Figure 4A). After two weeks of treatment, the disorientation of a few formed collagen fibers (score 0) was also found in SRP groups (Figure 4B). Moreover, in SRP+T and SRP+CU groups, less collagen fibers disorientation with little remodeling effect, and a moderate amount of collagen fibers, grouped in poorly cohesive beams (score 1), could be seen (Figure 4C,D). 

**Figure 5:**
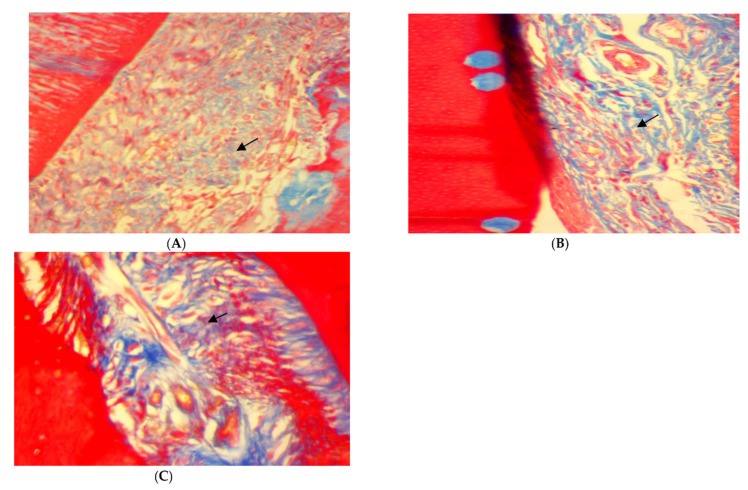
Histopathological features of rat periodontal tissue after four weeks of treatment show: (**A**) moderate amount of collagen fibers grouped in poorly cohesive beams (arrow) in SRP group. (**B**,**C**) The presence of well-oriented mature collagen fibers grouped in bundles with prominent remodeling effect (arrow) in SRP+T (**B**) and SRP+CU (**C**) groups (Trichrome ×400).

Moreover, Trichrome stain histopathological features of rat periodontal tissue in SRP group after four weeks of treatment showed a moderate amount of collagen fibers grouped in poorly cohesive beams (score 1) (Figure 5A), whereas the dominant presence of well-oriented mature collagen fibers grouped in bundles with a prominent remodeling effect (score 2) was seen in both SRP+T and SRP+CU groups (Figure 5B,C).

**Figure 6:**
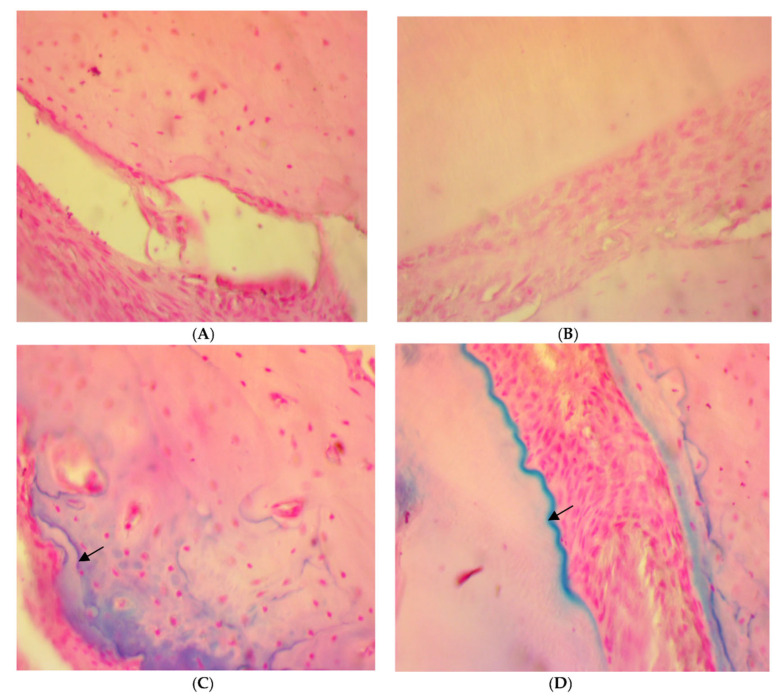
Histopathological features of rat periodontal tissue in SRP group (**A**) and SRP group+T (**B**) after four weeks of treatment show bone trabeculae is unstained and the lamellar organization of the matrix is evident. The surface of the bone trabeculae is labeled in blue, indicating a deposition of iron (arrow) in SRP+CU treatment (**C**,**D**) (Perls’ stain ×400).

On the other hand, a significant deposition of iron in the bone trabeculae was noticed in the SRP+CU group only (Figure 6C,D). The iron deposits appeared as blue linear bands in the bone matrix at the surface of the bone trabeculae. In contrast, no detectable iron with the Perls’ staining was found in the SRP group and SRP+T groups (Figure 6A,B).

### 2.2. Inflammatory Cells and Osteoclast Counts

A comparison between normal and EPD groups at baseline showed statistically significant differences between the number of total inflammatory cells and osteoclasts. A comparison between the groups that had received treatments at week 2 showed statistically significant differences between SRP group vs. SRP+T and SRP+CU in the total number of inflammatory cells and osteoclasts. Furthermore, a significant difference was detected in the number of osteoclasts when SRP+T and SRP+C were compared (*p* = 0.047). However, no significant differences were found between SRP+T and SRP+C in the total number of inflammatory cells (*p* = 0.99) (Table 1), whereas at week 4, a statistically significant difference was detected only in the total number of inflammatory cells when the SRP group was compared with SRP+T and SRP+CU (Table 1).

An intra-group comparison revealed a significant reduction in total inflammatory cell count and osteoclasts in SRP groups after four weeks of treatment as compared to two weeks. However, for SRP+T and SRP+CU groups, statistically significant differences were found in the total number of inflammatory cells in both SRP+T and SRT+CU groups after 4 weeks of treatment when compared to 2 weeks (Table 2).

## 3. Discussion

The prevalence and severity of periodontal disease can be decreased by mechanical plaque control and the selective removal or inhibition of pathogenic bacteria using either adjunctive systemic or local antimicrobials. The principle behind periodontal treatment is to repair or regenerate the tissues that have been lost or damaged. The conventional treatment of periodontal disease includes the mechanical removal of bacterial plaque (by scaling and root planing) to decrease the number of periodontal pathogens. However, SRP cannot lead to the complete removal of the periodontal pathogens and may be somewhat self-limiting because of bacterial pathogens’ ability to recolonize after treatment and the presence of inaccessible areas [5,6]. Thus, adjunctive antimicrobials, either in local or systemic form, have been used to overcome these limitations. Tetracycline has been used for subgingival administration in treatment of periodontitis [17].

In the present study, a significant reduction in inflammatory cells infiltration, osteoclast cells, and collagen fibers disorientation with angiogenesis and improvement of bone repair were found after two weeks in both the SRP+T and SRP+CU groups, which remained even after four weeks of treatment when compared to the SRP group. However, the differences after four weeks were not significant. This can be explained by the fact that both gels (tetracycline and curcumin) were applied for only ten days and their effect were lost when their applications stopped. These histological findings are in accordance with a study which reported that T is an antibiotic against most of the medically relevant bacteria, with osteoinductive ability to repair the infected bone after eight weeks [18]. Another study demonstrated that the T family are anti-inflammatory drugs with positive action for bone organization in the periodontal tissue of rats [19]. Furthermore, other studies reported that T inhibits collagenase and osteoclastic function [7,12], stimulates osteoblastic bone formation, regulates angiogenesis [20], and increases fibroblast insertion over radicular structure when associated with fibronectin [21].

Additionally, a study reported that after 7 and 15 days of local irrigation with T, the periodontal ligament was found to be intact and reorganized, with a predomination of oblique collagen fibers and fibroblasts with no inflammatory cells infiltration. The bone tissues showed organization with thick bone trabeculae and no signs of resorption, and the cementum surface did not present resorption areas [22]. It has been suggested that the anti-inflammatory properties of T can suppress the activity of polymorphonuclear cells, as it blocks the synthesis of prostaglandin E2 through the inhibition of phospholipase A2. These results are in accordance with the finding by the current study that the adjunctive use of T with SRP can decrease the inflammatory reaction and osteoclast number.

The use of medicinal plants in periodontitis treatment as antimicrobial natural products has attracted the attention of researchers as they offer a viable alternative adjunct therapy to SRP. Curcumin is one of the natural products that has been examined for this purpose. It has become known worldwide for its many health benefits and its clinical use as adjuvant to SRP in the treatment of chronic periodontitis [23,24,25]. In the present study, CU significantly reduced inflammation and osteoclast numbers and modulated collagen fiber and alveolar bone loss. This may be due to the CU modulating inflammatory activity by inhibiting nuclear factor kappa-B (NF-κB) activation and decreasing the osteoprotegerin (OPG)/soluble receptor activator of nuclear factor kappa-B ligand (sRANKL) ratio [26]. In addition, CU can reduce apoptosis and promote osteogenesis of the human periodontal ligament stem cells under oxidative stress, and might therefore have a potential clinical use with respect to tissue regeneration and bone osteogenesis [27].

The present results are similar to those reported by Xiao et al. [26] that indicated that CU significantly reduces gingival inflammation and modulates collagen fiber and alveolar bone loss in vivo. It was concluded that CU modulates inflammatory activity in rat periodontitis by inhibiting NF-κB activation and decreasing the OPG/sRANKL ratio induced by lipopolysaccharide (LPS) [26]. Moreover, another study also concluded that CU decreases alveolar bone loss in experimental periodontitis in rats via suppressing the expression of RANKL/RANK/OPG and possesses anti-inflammatory properties through the reduction of pro inflammatory cytokines TNF-a and IL-6 [28].

A study showed that clinically isolated *Porphyromonas gingivalis* obtained from chronic periodontitis patients was highly sensitive to CU, which stimulated quick fibroblast proliferation, re epithelialization, and scarring through the formation of thick well-organized bundles of collagen fibers [15]. Another study reported that the local administration of CU and chlorehexidine gels into EPD rats, induced by Porphyromonas *gingivalis* + ligature, resulted in a significant reduction in inflammatory cell infiltration and osteoclast cells numbers, with the improvement of periodontal ligament width associated with a reduction in serum levels of RANKL and IL-1β. The study concluded that CU was as effective as chlorhexidine in the treatment of EPD in rats. It reported that CU stops bone destruction related to periodontitis by regulating the RANKL and IL-1β markers level [29]. The current study also showed that CU gel was able to suppress the cytotoxic effects of PMNs and macrophages after 2 and 4 weeks of treatment. This finding is in line with previous studies regarding the anti-inflammatory properties and effects of CU that are believed to be due to the inhibition of NF-κB and κB-kinase activity [29,30,31]. In the same line, a study evaluated whether CU could ameliorate alveolar bone destruction in vivo and dissect its mechanism in vitro, the results demonstrating that CU dose-dependently reduced inflammatory bone loss in vivo by modulating RANKL-mediated osteoclast differentiation, activation, and function [32].

Our results also indicated that while SRP alone is still therapeutically beneficial, the CU gel yielded significant therapeutic improvement in total inflammatory cell count as compared to the control group at 2 and 4 weeks intervals. These results tended to remain statistically significant at later time intervals, implying continued improvements over time and with multiple applications of CU gel [13]. Recently, it has been reported that postbiotics, paraprobiotics, and probiotics can be used as adjunct to SRP and can reduce both clinical periodontal parameters and periodontopathogens [33,34].

It was reported that iron is critical for normal cellular differentiation of human periodontal ligaments cells. Iron deficiency or overload can have adverse effects on alveolar bone density [35]. Another study indicated that iron was possibly involved in the process of osteoblast cell differentiation [36]. In the present study, iron deposition in bone trabecula was detected in the SRP+CU group only, indicating that CU may contribute to promoting osteogenesis and osteoblast differentiation through altered iron level. Furthermore, another study found daily iron supplementation alongside CU may provide combined medical benefits without negatively influencing iron absorption [37]. Results suggested that iron deficiency anemia patients with chronic periodontitis have more periodontal breakdowns than patients with chronic periodontitis [38]. This study has limitations, including the short time period and not examining the clinical parameters of periodontitis. Furthermore, tartrate-resistant acid phosphatase stain has not been used for osteoclast counting, instead, osteoclast counting was performed by H&E stain carefully by an expert. Nevertheless, the results of the current study can be used as a base to further examine the repairing and osteogenesis effects of CU.

## 4. Materials and Methods

### 4.1. Rats and Housing

A total of 32 male Wister-albino rats, aged 2–3 months and weighing 250–300 g, were used in this study; the study followed the principles of laboratory animal care (NIH publication 85–23, 1985) [39] while they were cared for in the animal house of the College of Medicine, Hawler Medical University. The study protocol was approved by the Ethics Committee of the College of Dentistry/Hawler Medical University. All rats were allowed to adapt to the housing conditions for one week prior to the commencement of the trial. Four rats were housed in each wire cage and maintained on a 12-h light/dark cycle at 20 ± 5 °C and 20–30% humidity. The animals were kept in standard room conditions and fed with a standard rat chow and allowed to drink water ad libitum.

### 4.2. Induction of Experimental Periodontitis

General anesthesia was applied via intramuscular injection of ketamine (40 mg/kg of b.w). The animals were placed on a proper operating table, which allowed open-mouth maintenance of the rats to facilitate access to the teeth. In total, there were 4 rats with healthy gingiva and the remainder had experimental periodontitis (EPD), which was induced by placing 3.0 sterile black braided silk threads around the cervix of the mandibular incisors and keeping them in place for two weeks [4]. This ligature acted as a gingival irritant, promoted the accumulation of plaque and subsequently development of periodontal disease. Ligatures control and checking was performed daily, and if any ligature had been lost or become loose, it was replaced.

### 4.3. Experimental Design

The 32 animals were randomly assigned into five groups as follows:

Group 1. Healthy control, included four rats with no experimental periodontitis (healthy control group).

Group 2. Positive control EPD, included four rats with EPD (EDP group).

Group 3. Included eight rats with experimental periodontitis EPD treated with SRP alone (SRP group).

Group 4. Included eight rats with experimental periodontitis treated with SRP and T gel (SRP+T group).

Group 5. Included eight rats with experimental periodontitis treated with SRP and curcumin gel (SRP+CU group).

**Figure 7:**
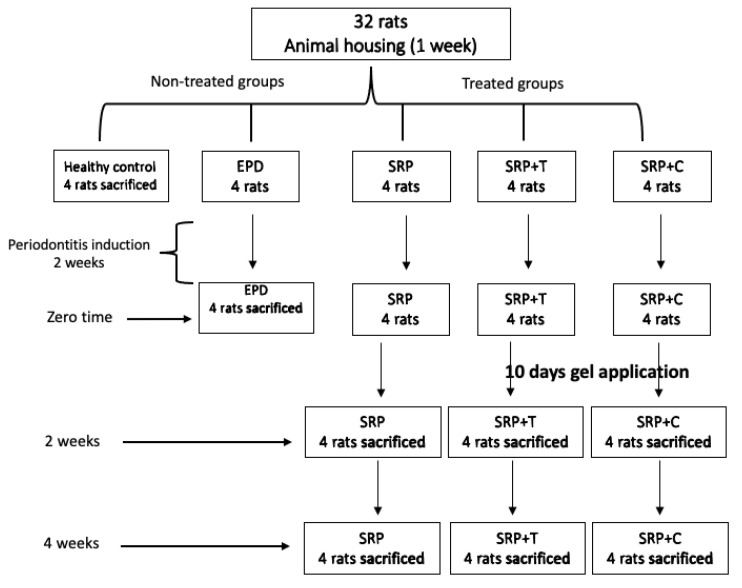
Excremental design of the studied groups.

In the last three treated groups, rats received SRP using manual 1–2 mini-five Gracey curettes (Hu-Friedy Company, Chicago, IL, USA), followed by subgingival application of gel for 10 days in SRP+CU and SRP+T groups. The local application of gel was performed immediately after SRP and repeated twice daily for 10 days using a plastic syringe with blunt needle (Figure 7).

### 4.4. Curcumin Gel Composition and Preparation

In SRP+CU group, rats received 12.5 μg/mL of CU gel immediately after SRP [14]. CU gel contained CU powder: 95% CU (Bulk Supplements Pure CU 95% Natural Turmeric Extract Powder), Potassium sorbate (Analitik Kimya Ve Lab. Cih. San. Tic. Ltd. Sti. Istanbul, Turkey), Propylene glycol (Pharmaco-Aaper, Bengaluru, India), Metalose 90SH 10,000 (Shin-Etsu Chemical Co., Chiyoda, Japan) and purified water. The muco-adhesive gel was prepared by Awa Medica Company, Hawler, Kurdistan Region, Iraq.

CU gel was prepared by:

- Dissolving and mixing 12.50 μg of CU with 100 mL propylene glygol and 25.00 mg potassium sorbate.
- Dissolving 4 mg of Metalose 90SH 10,000 in 850 mL purified water.
- The solutions from a and b were mixed and the volume was made up with water; finally, the mixing was continued until a homogenous gel was formed.

### 4.5. Tetracycline Gel Composition and Preparation

Tetracycline gel was prepared by:

- Dissolving and mixing 6 μg of T with 100 mL propylene glygol and 25.00 mg of potassium sorbate.
- Dissolving 4 mg of Metalose 90SH 10,000 in 850 mL purified water.
- The solutions from a and b were mixed and the volume was made up with water; finally the mixing was continued until a homogenous gel was formed. Tetracycline gel was also prepared by Awa Medica Company, Hawler, Kurdistan Region, Iraq.

### 4.6. Histological Evaluation

The animals in control group were anesthetized and sacrificed by cervical dislocation after 7 days of animal housing adaptation, immediately after ligature-induced periodontitis in EPD group (zero time), 2 and 4 weeks after SRP in SRP group, and 2 and 4 weeks after SRP and subgingival application of CU and T gels in SRP+CU and SRP+T groups (Figure 7). First, the mandibles were resected and bisected from both sides posterior to the incisor and then processed to prepare H&E stained tissue sections. The sample was fixed for 72 h in 10% neutral buffered formalin and then decalcified in a 1/1 mixture of 8% formic acid and 8% hydrochloric acid for 3 weeks. Following decalcification, the tissue sample was trimmed and dehydrated through successive baths of Isopropyl alcohol (70%, 90%, 95%, and 100%), clarified in xylene, and embedded in paraffin wax [40]. Multiple longitudinal sections (5 sections) in labio-lingual direction in gingival connective tissue were trimmed from each paraffin block at 5 µm thickness with a rotary microtome (Leica RM2135). Later, tissue sections were stained with hematoxylin and eosin (H&E) for histological examination.

### 4.7. Histo-Morphometric Assessment

Quantitative assessment for inflammatory reaction in the periodontal tissue at the lingual side was performed (at 400-fold magnification) under a digital light microscope equipped with an image analyzing system (Motic, ToupTek, ToupView, ×86, 3.7.4183, and 2014). Each captured image was divided by a grid containing 16 squares. Neutrophils, lymphocytes, and macrophages were counted and the mean number for each group calculated.

### 4.8. Evaluation of Inflammatory Reaction

Severity of inflammatory reaction was obtained by calculating the number of inflammatory cells within magnification power of ×400 for all studied groups as follows [16]:

Score 0: none or few inflammatory cells, from 0 to less than 5, no inflammatory reaction.

Score 1: the inflammatory cells number from 5 to less than 25, mild inflammatory reaction.

Score 2: the inflammatory cells number from 25 to less than 125 cells, moderate inflammatory reaction.

Score 3: the inflammatory cells number more than or equal to 125 cells, severe inflammatory reaction.

Furthermore, the volume of the granulation tissue, degree of angiogenesis, and fibrous tissue deposition were evaluated. The degree of change was estimated semi-quantitatively, by forming a score based on the extent of the change as follows [41]:

Score 0: Indicated poor parameter expression.

Score 1: Indicated moderate parameter expression.

Score 2: Indicated strong parameter expression.

Finally, osteoclasts cells were identified as having multiple nuclei with ruffled border and granular cytoplasm in H&E stain sections. The sum of osteoclasts on the surface of alveolar bone was calculated in each section for each group along a distance of 130 μm from the alveolar crest.

### 4.9. Special Stains

Perls’ stain was used to detect iron deposition in bone trabeculae. Furthermore, appropriate tissue sections were stained with Masson’s Trichrome. The amount and nature of the deposited collagen fibers (seen at Trichrom stained samples) were evaluated as follows [38]:

Score 0: Indicated the presence of early, immature collagen fibers, mostly in the reticular arrangement.

Score 1: Indicated the presence of moderate amount of collagen fibers, grouped in poorly cohesive beams.

Score 2: Indicated dominant presence of mature collagen fibers, grouped in bundles.

### 4.10. Statistical Analysis

The values of studied independent variables were presented as mean ± standard deviation. *t* test was used to make a comparison between two groups. One-way ANOVA test was used to determine the significance of differences among the three experimental treated groups, followed by Tukey’s honest significant difference (HSD) test for multiple pairwise comparisons. Significance level was defined at *p* < 0.05. All statistical methods were performed by using SPSS software (version 25, IBM, Chicago, IL, USA).

## 5. Conclusions

The results from this study suggest that the adjunctive use of CU with SRP can reduce inflammation and alveolar bone resorption. The anti-inflammatory effect of CU is comparable to that of T. Furthermore, CU has potential osteogenesis and healing effects. Further studies with a longer follow-up period and examination of the exact mechanism of the osteogenesis effect and healing process of CU are highly recommended. Moreover, clinical trial studies on the adjunctive use of prostbiotics to SRP as a newly discovered agent are highly recommended.

## Figures and Tables

**Table 1:** Total inflammatory cell count in normal and experimental periodontitis groups, showing comparison of the three treated groups after 2 and 4 weeks.

Time	Groups	Comparison	Total Inflammatory Cell Count Mean ± S.D.	*p*-Value	Osteoclast	*p*-Value
-	Normal	Normal vs. EPD	2.33 ± 0.58	0.001 *	0	0.001 *
	EPD		31.00 ± 1.00		4.67 ± 0.58	
**2 Weeks**	SRP		20.33 ± 2.52		2.67 ± 0.58	
	SRP+T	SRP vs. SRP+T	5.00 ± 0.00	**0.001 ^†^**	1.67 ± 0.58	**0.047 ^†^**
	SRP+CU	SRP vs. SRP+CU	5.00 ± 0.00	**0.001 ^†^**	1.33 ± 0.58	**0.026 ^†^**
		SRP+T vs. SRP+CU		0.99 †		**0.047 ^†^**
**4 Weeks**	SRP		5.00 ± 0.00		1.67 ± 0.58	
	SRP+T	SRP vs. SRP+T	3.33 ± 0.58	**0.002 ^†^**	1.67 ± 0.58	0.99 ^†^
	SRP+CU	SRP vs. SRP+CU	2.67 ± 0.58	**0.0008 ^†^**	1.00 ± 0.00	0.116 ^†^
		SRP+T vs. SRP+CU		0.48 ^†^		0.116 ^†^

EPD: Experimental periodontitis; SRP: scaling and root planning; SRP+T: scaling and root planing and tetracycline; SRP+CU: scaling and root planing and curcumin; *: *t* test; ^†^: One-way ANOVA.

**Table 2:** Comparison between the experimental periods for the treated groups.

Groups	Time	Total Inflammatory Cell Mean ± SD	Osteoclasts Mean ± SD
SRP	2 Weeks	20.33 ± 2.51	2.67 ± 0.58
	4 Weeks	5.00 ± 0.00	1.67 ± 0.58
	*p* value *	0.005 *	0.047 *
SRP+T	2 Weeks	5.00 ± 0.00	1.67 ± 0.58
	4 Weeks	3.33 ± 0.58	1.67 ± 0.58
	*p* value *	0.007 *	1.00 *
SRP+CU	2 Weeks	5.00 ± 1.00	1.33 ± 0.58
	4 Weeks	2.67 ± 0.58	1.00 ± 0.00
	*p* value *	0.007 *	0.37 *

SRP: scaling and root planing; SRP+T: scaling and root planing and tetracycline; SRP+CU: scaling and root planing and curcumin; *: *t* test.

## Data Availability

The data presented in this study are available on request from the corresponding author.
